# Separating N_2_O production and consumption in intact agricultural soil cores at different moisture contents and depths

**DOI:** 10.1111/ejss.13363

**Published:** 2023-04-27

**Authors:** Erik S. Button, Karina A. Marsden, Philip D. Nightingale, Elizabeth R. Dixon, David R. Chadwick, David L. Jones, Laura M. Cárdenas

**Affiliations:** ^1^ School of Natural Sciences Bangor University Bangor Gwynedd UK; ^2^ Plymouth Marine Laboratory, Prospect Pl, Marine Biogeochemical Observations Plymouth Devon UK; ^3^ Rothamsted Research North Wyke, Net Zero and Resilient Farming Okehampton Devon UK; ^4^ Centre for Sustainable Farming Systems, Food Futures Institute Murdoch Western Australia Australia

**Keywords:** denitrification, diffusion coefficient, isotope pool dilution, nitrogen cycling, sulphur hexafluoride

## Abstract

Agricultural soils are a major source of the potent greenhouse gas and ozone depleting substance, N_2_O. To implement management practices that minimize microbial N_2_O production and maximize its consumption (i.e., complete denitrification), we must understand the interplay between simultaneously occurring biological and physical processes, especially how this changes with soil depth. Meaningfully disentangling of these processes is challenging and typical N_2_O flux measurement techniques provide little insight into subsurface mechanisms. In addition, denitrification studies are often conducted on sieved soil in altered O_2_ environments which relate poorly to *in situ* field conditions. Here, we developed a novel incubation system with headspaces both above and below the soil cores and field‐relevant O_2_ concentrations to better represent *in situ* conditions. We incubated intact sandy clay loam textured agricultural topsoil (0–10 cm) and subsoil (50–60 cm) cores for 3–4 days at 50% and 70% water‐filled pore space, respectively. ^15^N‐N_2_O pool dilution and an SF_6_ tracer were injected below the cores to determine the relative diffusivity and the net N_2_O emission and gross N_2_O emission and consumption fluxes. The relationship between calculated fluxes from the below and above soil core headspaces confirmed that the system performed well. Relative diffusivity did not vary with depth, likely due to the preservation of preferential flow pathways in the intact cores. Gross N_2_O emission and uptake also did not differ with depth but were higher in the drier cores, contrary to expectation. We speculate this was due to aerobic denitrification being the primary N_2_O consuming process and simultaneously occurring denitrification and nitrification both producing N_2_O in the drier cores. We provide further evidence of substantial N_2_O consumption in drier soil but without net negative N_2_O emissions. The results from this study are important for the future application of the ^15^N‐N_2_O pool dilution method and N budgeting and modelling, as required for improving management to minimize N_2_O losses.

**Highlights**

- Explores how N_2_O diffusion, production and consumption vary with soil depth and soil moisture.
- A novel and more field‐relevant system was developed to incubate intact top‐ and subsoil cores.
- Diffusion was driven by moisture and N_2_O consumption and production were highest in drier soil.
- This new system can separate N_2_O processes occurring at depth whilst replicating field conditions.

## 1 INTRODUCTION

Nitrous oxide (N_2_O) exchange between the soil and atmosphere has received significant attention in recent decades because of its prominent role in climate change and atmospheric ozone depletion (e.g., Jia et al., 2019). More than half of global agricultural greenhouse gas emissions are from N_2_O, resulting from N inputs to soil, including fertilizer and manure application (direct) and denitrification following leaching and atmospheric deposition of nitrogen (N; indirect) (Clough et al., 2005; Jia et al., 2019). There are several pathways and processes (both biotic and abiotic) that produce and consume N_2_O in soils (see Butterbach‐Bahl et al., 2013), however, nitrification and denitrification are widely considered the major N_2_O producing processes. Under suboxic conditions, the production of atmospheric N_2_O is primarily governed by microbial incomplete denitrification in the soil, where N_2_O is produced from nitrate (NO_3_^−^) under partially anaerobic conditions (Table S1; Diba et al., 2011). Nitrification (Table S1) is an aerobic process, and some studies have shown it can be the dominant N_2_O producing process (e.g., Liu et al., 2016; Zhang et al., 2016), especially where soil aeration is sufficient (35%–60% water‐filled pore space [WFPS]; Bateman & Baggs, 2005). However, denitrifiers can also consume N_2_O (i.e., complete denitrification; Table S1) to produce inert dinitrogen (N_2_) gas (Diba et al., 2011), which constitutes 78% of the Earth's atmosphere. Typically, N_2_ is the major end product of denitrification, where the soil moisture is greater than 80% WFPS (Giles et al., 2017) as it is performed by facultative anaerobic microorganisms (Butterbach‐Bahl et al., 2013). This process is often masked by greater production rates and is mostly measured only when the consumption rate exceeds the production rate (i.e., net negative emissions; Chapuis‐Lardy et al., 2007; Schlesinger, 2013). Measuring the consumption of N_2_O directly (e.g., by N_2_ flux) is challenging against a very high atmospheric background (Clough et al., 2006; Wen et al., 2016; Yang et al., 2011). In addition, the heterogeneity of N_2_O processes in the soil and their measurement can lead to high error when data is scaled (Groffman et al., 2006). Accurately measuring N_2_O consumption is important for modelling and prediction of future soil N budgets, for which N_2_O is the most poorly constrained term, due to the abovementioned inherent challenges (Almaraz et al., 2020; Blagodatsky & Smith, 2012; Boyer et al., 2006).

The balance between gross production and consumption of N_2_O in agricultural soil is complex, being influenced by a range of environmental factors (e.g., temperature, moisture, O_2_ content; Chapuis‐Lardy et al., 2007), soil characteristics (e.g., pH, mineral N content, porosity, organic matter content, soil depth; Chapuis‐Lardy et al., 2007; Clough et al., 2005; Stuchiner & von Fischer, 2022a) and management practices (e.g., fertilizing regime, tillage, irrigation; Khalil et al., 2002; Wang et al., 2018).

The consumption of N_2_O is stimulated by anaerobic conditions (high WFPS) due to the sensitivity of the metallo‐enzyme, N_2_O reductase, to O_2_ (Richardson et al., 2009). Thus, extensively waterlogged soils, such as peat‐ and wetlands represent the greatest N_2_O sinks globally (Schlesinger, 2013). Low mineral N content is also thought to be important for N_2_O consumption, because nitrate (NO_3_^−^) outcompetes N_2_O as a terminal electron acceptor (Chapuis‐Lardy et al., 2007). However, N_2_O consumption has been found to coincide with low WFPS in both fertilized (<50% WFPS; Khalil et al., 2002) and unfertilised soil (5%–20% WFPS; Wu et al., 2013). Here, anaerobic conditions may exist in microsites heterogeneously distributed throughout the soil profile of free‐draining soils, within soil aggregates (even in dry aerobic soil; Sexstone et al., 1985) or can be caused by localized respiration hot spots that deplete O_2_ (Clough et al., 1999; Hill & Cardaci, 2004; Van Cleemput, 1998). Therefore, N_2_O produced in the soil is not necessarily consumed in the same location but may diffuse to another site in the soil, may be lost to the atmosphere or groundwater (Shcherbak & Robertson, 2019), or become entrapped in the soil (Clough et al., 1999). In addition, aerobic consumption of N_2_O is possible, where N_2_O is used as an electron acceptor when NO_3_^−^ is limited (Chapuis‐Lardy et al., 2007; Wang et al., 2018). To understand these processes in a meaningful way, the physical diffusion and the gross N_2_O production and consumption rates need to be separated from each other.

N_2_O processes occurring deeper in the soil have received less attention but are important in understanding the balance between N_2_O production and consumption (Almaraz et al., 2020; Clough et al., 2005; Jahangir et al., 2012). The movement of N_2_O to the soil surface is predominantly via passive diffusion through air‐filled pores in the soil. The concentration of N_2_O at depth is frequently higher than near the soil surface because of lower diffusivity (Balaine et al., 2013; Currie, 1984; Davidson et al., 2004; Dong et al., 2013; Fujikawa & Miyazaki, 2005; Laughlin & Stevens, 2002; van Bochove et al., 1998; Van Groenigen et al., 2005; Wang et al., 2018; Zona et al., 2013). This lag between production and surface emission is supported by a ^15^N‐labelled experiment by Clough et al. (1999), where it took 11 days for N_2_O produced at 80 cm to first reach the soil surface and 6% remained in the soil even after 38 days (i.e., entrapment). Soil conditions restricting N_2_O diffusion, thereby increasing its residence time in the soil, can increase its consumption (Chapuis‐Lardy et al., 2007; Clough et al., 2005; Neftel et al., 2007). The generally higher rate of N_2_O consumption and production in the topsoil is a reflection of the greater microbial abundance and activity (Van Beek et al., 2004; van Bochove et al., 1998; Wang et al., 2018) than in subsoils, but considerable N_2_O production and consumption can also occur in the subsoil if conditions allow (Clough et al., 1999; Shcherbak & Robertson, 2019). In addition, an understanding of the relationship between diffusion and N_2_O emissions is lacking (Balaine et al., 2013), especially in intact deep soil (Chamindu Deepagoda et al., 2019). Therefore, understanding the balance of N_2_O production and consumption between topsoil and subsoil depths under different soil conditions and their relation to diffusion is needed to best predict N_2_O surface emissions for modelling the global N budget (Almaraz et al., 2020; Blagodatsky & Smith, 2012; Boyer et al., 2006).

Understanding N_2_O mechanisms in the soil is important for more accurate modelling and N budgeting, and to support emerging attempts to minimize N_2_O losses from soil. Chamindu Deepagoda et al. (2019) found a range of relative gas diffusivity rates which lowered N_2_O emissions that could be monitored and maintained by land users. Stuchiner and von Fischer (2022a) recently demonstrated a case of Increased Consumption and Decreased Emissions (coined ICDE) of N_2_O via promotion of anoxia from relieving the C‐limitation to the microbial community.

The ^15^N_2_O pool dilution method is a relatively new method used by Yang et al. (2011), Yang and Silver (2016), and Wen et al. (2016, 2017) to determine the gross production and consumption of N_2_O. The method, where isotopically enriched ^15^N_2_O is injected into a closed system and the disappearance of the label is measured over time, is currently the only method for field measurement of gross N_2_O emission and uptake under undisturbed conditions (Almaraz et al., 2020). This method can also be applied to the incubation of soil cores, as performed by Wen et al. (2016) and Stuchiner and von Fischer (2022a), which allows for the incubation of soil cores taken from below the surface. An inherent assumption of the ^15^N_2_O pool dilution method is that the ^15^N_2_O that diffuses into the soil mixes evenly with soil‐derived N_2_O. Wen et al. (2016) compared the pool dilution method with a gas‐flow core method and found it to underestimate gross N_2_O production and consumption. As a result of the use of a closed static system in previous applications of the method, the diffusion and mixing of the labelled gas with soil pores is less likely to occur, which means that gross N_2_O production and consumption may be underestimated. Therefore, a system in which the mixing of the label with the soil pores is improved will result in greater accuracy of the pool dilution approach.

In this study, we used a novel open dual headspace system with field‐relevant O_2_ concentrations to incubate intact sandy clay loam agricultural topsoil and subsoil cores. This system was developed to answer the following question: *does the balance between soil N*_*2*_ *O production and consumption differ between soil depths and moisture contents in intact agricultural soil cores?* Following the ^15^N‐N_2_O pool dilution (Wen et al., 2016; Yang et al., 2011) and Currie method (Currie, 1960) with SF_6_ as a conservative tracer, the relative diffusivity (*D*_s_/*D*_0_), net N_2_O emission, and gross N_2_O emission and uptake rates were measured. We hypothesised that, (i) the rate of diffusion would decrease with soil depth and wetness due to greater soil density and lower porosity; (ii) despite higher N_2_O and lower O_2_ concentrations deeper in the soil, consumption of N_2_O will be greater in the more microbially active topsoil; and (iii) a WFPS above the critical level (ca. ≥60%; Bateman & Baggs, 2005) will increase N_2_O consumption, whereas at a lower WFPS, N_2_O consumption will be minimal.

## 2 MATERIALS AND METHODS

### 2.1 Soil collection and characterization

Sandy clay loam textured freely draining arable soil was collected from Abergwyngregyn, North Wales (53°14′29″ N, 4°01′15″ W) in February 2020. The soil is classified as a Eutric Cambisol (WRB) or Typic Hapludalf (US Soil Taxonomy) and has a crumb structure because of high levels of earthworm bioturbation. This soil was chosen because it is a globally extensive temperate soil type and is common in agricultural production (WRB, 2014). Prior to collection, the field had been used for winter wheat (*Triticum aestivum*) production. Soil was collected from 6 randomly selected locations within the field from the topsoil (0–10 cm) and subsoil (50–60 cm), which were retained as 6 independent replicates. The latter soil depth was from below the plough layer and the field had no history of subsoiling. The latter depth was chosen as it was representative of the B horizon, assumed to be ‘undisturbed’ from mechanical soil management and provided enough distinction in soil characteristics from the topsoil cores. Two disturbed soil samples and three intact soil cores (using stainless steel rings of 53 mm outside diameter × 50 mm height, 104 cm^3^ volume; steel from Complete Stainless Ltd., Glasgow, UK) were collected from each hole at each depth, excluding spare cores used for soil characterization. Soil cores were collected by lightly hammering in the steel rings at the appropriate soil depth and retrieving them, when the entire volume was filled, by carefully digging them out. The soil cores were then placed in plastic bags (but not sealed) in the field and stored at <5°C prior to use.

One of the sets of three soil cores per depth and hole were removed from their metal core rings, weighed and oven‐dried (105°C, 24 h) immediately after collection. The dry bulk density was determined by dividing the dry weight by the soil volume. WFPS was determined using the volumetric water content, particle density, and bulk density, using the following equation:

(1) $$\text{WFPS}=\frac{B_{d}∙M_{c}}{1-\frac{B_{d}}{P_{d}}}∙100,$$

where the *B*_d_ is the dry bulk density (g cm^−3^) and *M*_c_ is the moisture content (g g^−1^) and their product is the volumetric water content (cm^3^ cm^−3^). *P*_d_ is the particle density, assumed at 2.65 g cm^−3^. 1 − *B*_d_/*P*_d_ is the total porosity (cm^3^ cm^−3^).

5 g replicates of soil were extracted using 0.5 M K_2_SO_4_ at a ratio of 1:5 (w/v) on the same day the soil was collected. These were shaken at 200 rpm for 30 min and then centrifuged (14,000*g*, 10 min). The supernatant was then removed and frozen for ammonium (NH_4_^+^) and NO_3_^−^ content determination by colourimetry, according to Mulvaney (1996) and Miranda et al. (2001), respectively, with a PowerWave XS Microplate Spectrophotometer (BioTek Instruments Inc., Winooski, VT). Dissolved organic C and N in the extracts was determined using a Multi N/C 2100/2100 analyser (AnalytikJena AG, Jena, Germany). Dissolved organic N was determined by subtracting inorganic N (NO_3_^−^ and NH_4_^+^) from the total dissolved N. Soil EC and pH in water were determined in a 1:5 ratio (w/v) using a Jenway 4520 conductivity meter and a Hanna 209 pH meter (Hanna Instruments Ltd., Leighton Buzzard, UK), respectively. A summary of the initial soil properties is provided in Table 1.

**TABLE 1:** Properties of the Eutric Cambisol topsoil (0–10 cm) and subsoil (50–60 cm) used for the study.

Properties	Topsoil	Subsoil
	0–10 cm	50–60 cm
Sand (%)^a^	62.9 ± 0.7	67.2 ± 6.5
Silt (%)^a^	16.2 ± 1.3	14.9 ± 3.1
Clay (%)^a^	20.9 ± 1.0	17.9 ± 4.1
Dry bulk density (g cm^−3^)	1.11 ± 0.06	1.26 ± 0.04
Porosity (%)	55.7 ± 0.8	53.0 ± 3.5
Organic C (g C kg^−1^)	27.8 ± 1.3	7.4 ± 1.0
Total N (g N kg^−1^)	3.4 ± 0.1	1.5 ± 0.1
C:N ratio	8.1 ± 0.1	4.8 ± 0.3
pH_H2O_	6.8 ± 0.06	6.8 ± 0.03
EC (μS cm^−1^)	1198 ± 126	657 ± 102
Extractable NH_4_^+^ (mg N L^−1^)	0.08 ± 0.02	0.09 ± 0.03
Extractable NO_3_^−^ (mg N L^−1^)	41.1 ± 6.0	22.4 ± 5.2
Dissolved organic C (mg C L^−1^)	12.6 ± 1.3	4.3 ± 1.8
Dissolved organic N (mg N L^−1^)	4.9 ± 2.1	0.6 ± 0.4
Soil microbial biomass (mg C kg^−1^)	74.0 ± 3.7	42.9 ± 1.4

*Note*: Values represent means ± SEM (*n* = 4) and values are expressed on a dry soil weight equivalent where appropriate.

^a^ Data from Sanchez‐Rodriguez et al. (pers. comm.), *n* = 4.

### 2.2 Experimental system

**FIGURE 1:**
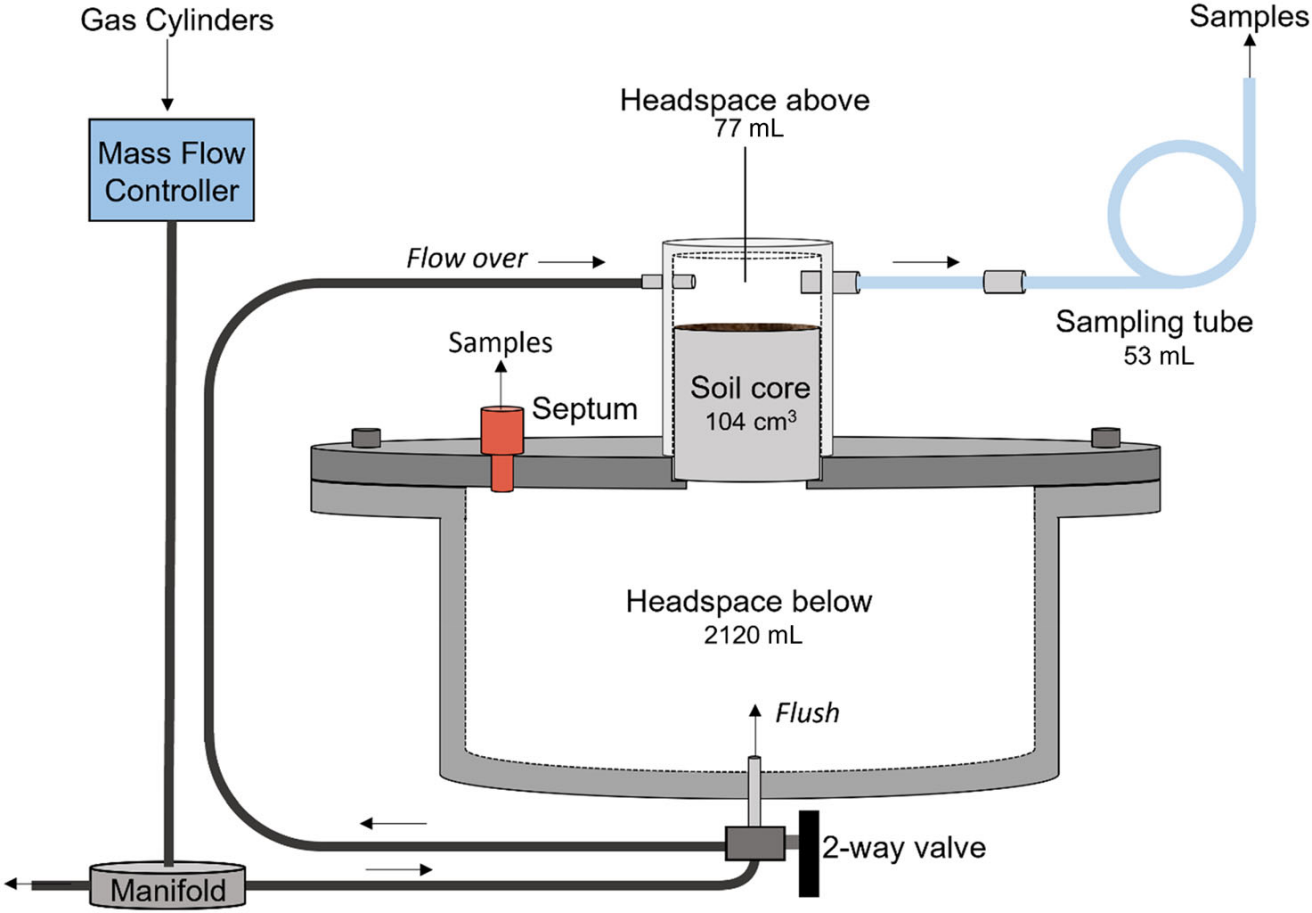
The dual‐headspace system used for incubating the soil cores in this study. The system can be placed in two different modes, ‘flush’ and ‘flow over’. The former is where air flow from the gas cylinders is directed to enter via the headspace below the core, while the latter directs this air via the headspace above the soil core. All dimensions, materials and a photograph of the system can be found in the Supplementary Information (S1).

A specialized gas‐flow‐soil‐core incubation system (DENitrification Incubation System [DENIS]; Cárdenas et al., 2003), allowing controlled environmental condition control (including O_2_ concentration and temperature), was adapted for this study using custom‐made lids used by Boon et al. (2013). The system, with 12 large individual stainless steel chambers (2120 mL), was modified to hold 53 mm wide soil cores with a lid and septum for direct gas application and sampling from a small headspace (77.2 mL) with a 3 m (4.8 mm ID, 53.4 mL) sampling tube (Figure 1). Details of the DENIS modification and a photograph are provided in the Supplementary Information (S1, Figure S1).

The gas flow from the O_2_ and N_2_ (see ratios in 2.4) cylinders into the system was adjusted via mass flow controllers (MFC) to achieve the desired flow rate and O_2_ concentration, and then split evenly into each of the 12 incubation vessels via a manifold. A valve (Figure 1) enabled flow to be either directed to enter the large headspace below the intact soil cores (‘flush mode’) or to enter the small headspace on top of the intact soil cores (‘flow over mode’). In both modes the gas exited via the sampling tube. The MFC was calibrated for all gases used in the experiment by measuring the flow 5 times at 10 flow rate settings with a bubble meter.

In this study, two gases were used to generate the ‘flush’ and ‘flow over’ the intact soil cores (Figure 1): an ECD‐Grade N_2_ cylinder and a grade zero O_2_ cylinder (BOC; Linde plc, Guildford, UK). The N_2_ cylinder and a compressed air line that was used for the ‘flow over mode’ both had SF_6_ concentrations below atmospheric levels (i.e., <10 ppt). During pilot studies, we discovered that the SF_6_ concentration in the O_2_ cylinder was surprisingly high (ca. 6 ppb), which is about three orders of magnitude greater than the concentration of atmospheric SF_6_ (10.6 ppt). Therefore, we decided to use this as our source of SF_6_ for the incubation.

^15^N labelled N_2_O was generated specifically for this experiment using the ammonium sulphate method described by Laughlin et al. (1997). This generates N_2_O and N_2_ at the same ^15^N enrichment as the ammonium sulphate. The generated N_2_O and N_2_ were collected in evacuated exetainers (Labco Ltd., Lampeter, UK). N_2_ was removed using the cryotrapping loops in a Sercon trace gas analyser (TG2, Sercon Ltd., Crewe, UK) so that the N_2_O was trapped while the N_2_ was flushed to waste. Once the N_2_ had been removed, N_2_O was collected in a Tedlar® gas sample bag from the outlet of the TG2. The contents of the Tedlar® bag were analysed for N_2_O and N_2_ concentration and enrichment using a Sercon trace gas analyser and Sercon 20:22 isotope ratio mass spectrometer (Sercon Ltd., Crewe, UK).

### 2.3 Soil core preparation and installation

Soil cores from both depths were brought to either 50% or 70% WFPS for the incubation experiment. These WFPS were chosen as they are either side of the 60% WFPS threshold for N_2_O production and N_2_O produced is likely to be underpinned by different processes (Bateman & Baggs, 2005). Soil cores were brought to the desired weight for attaining a WFPS of 50% or 70% (*n* = 6 each) by adding distilled water (70% WFPS) or air‐drying the approximate field moist soil (50% WFPS). To calculate the difference in moisture content (Δ*M*_c_) for achieving for the required WFPS (50% or 70%) in the incubation, the required WFPS level (WFPS_R_; %) was multiplied by the total pore space volume (PS_v_; cm^3^) as demonstrated in Equation (2). The moisture content of the core (*M*_cc_; cm^3^) was then subtracted to obtain the difference in the soil core moisture to achieve the required WFPS.

(2) $$\Delta M_{c}=\frac{{\text{WFPS}}_{R}∙{\mathrm{PS}}_{v}}{100}-M_{\mathrm{cc}}.$$

The water required to reach the desired WFPS level in the cores was pipetted onto the surface of the cores 24 h before installation in the incubation system. Where this water did not immediately infiltrate, it was done in stages so all the water was pipetted and it did not run down the sides of the core. Cores that required WFPS level reduction were air‐dried and subsequently adjusted with additional water if they overshot the target (as described above). Cores that had not lost enough weight after air‐drying overnight to meet the required WFPS were further dried in an incubator at 40°C (see Supplementary Information S3, for more information). Once all the cores had attained the target WFPS, they were installed randomly in the system (Figure 1). The inside edges of the top of the soil cores (ca. 2–4 mm) were carefully sealed with silicone grease to ensure no edge related diffusion effects. This was also done on the bottom of the soil cores, where drying had caused cores to slightly (<1 mm) shrink away from the metal core ring. A circular nylon mesh was placed in the lid groove before the cores were installed to prevent the soil from falling into a large headspace. The cores were then lightly tapped into the steel lids of the large headspaces of the incubation vessels using a mallet. The inside walls of the small headspace chambers and where they met the large headspace lids were also greased with silicone to ensure an airtight fit. This was confirmed by measuring gas flow through all 12 cores using a bubble flow meter.

### 2.4 Soil core incubation

Soil cores were incubated in the dark, and the temperature in the laboratory was kept constant at 22°C for the 4–5 day incubation (depending on soil depth). As an acclimatization period, the soil cores were put into ‘flush mode’ at a flow rate of 5 mL min^−1^ core^−1^ for ca. 18 h with an SF_6_‐containing (see Section 2.2) O_2_:N_2_ mixture. This mix was 20.9:100 and 13:100 O_2_:N_2_ for the 0–10 and 50–60 cm cores, respectively. The O_2_ content of the mix was chosen by a fitted trend of a similar soil profile (Figure S3). The acclimatization period allowed the air‐filled pore space to attain an air mix representative of the soil core depths and for the accumulation of a reservoir of SF_6_ tracer gas in the headspace below the soil core.

After the ‘flush mode’, the gas flow was momentarily stopped and the (high SF_6_) O_2_ cylinder was exchanged for a (ambient‐SF_6_) compressed air cylinder and the flow adjusted to maintain the same O_2_:N_2_ ratio. The flow was changed to ‘flow over mode’ by switching the valve below the large headspace to divert the gas to flow over the small headspace (Figure 1) and resumed at the same rate (ca. 5 mL min^−1^ vessel^−1^) for the rest of the experiment. The vessels were left for ca. 4 h to remove the high SF_6_ gas concentrations in the above core headspace from the ‘flush mode’. 60 mL of 30 atom% containing 85 and 100 ppm ^15^N‐isotopically labelled N_2_O was then syringe‐injected into the 0–10 cm and 50–60 cm core large headspace vessels (below the intact soil cores) via the septum (Figure 1), achieving a ^15^N_2_O headspace concentration below the soil core of 2.4 and 2.8 ppm, respectively. These represent the in situ concentrations of N_2_O at the same field site between the two depths (ca. 30 cm; Figure S4). The flow rate was tested daily three times per core after sampling using a bubble meter, and these specific flow rates were used to calculate the fluxes.

### 2.5 Gas sampling and analysis

Approximately 30 min after injection of the ^15^N_2_O into the headspace below the intact soil cores, the large headspace was assumed to be mixed and the initial ‘*t* = 0’ SF_6_ (10 mL) and mass spectrometry (duplicate 12 mL) samples were taken using separate gas‐tight 20 mL polypropylene syringes. The samples were assumed to be representative of the large headspace by filling and emptying the syringe three times into the headspace before a gas sample was taken. SF_6_ samples were analysed immediately, while the duplicate samples for mass spectrometry were injected directly into 12 mL pre‐evacuated (flushed with Helium and doubly evacuated) Exetainers® (Labco Ltd., Lampeter, UK). Below core headspace samples were taken daily for SF_6_ analysis. Samples from the headspace below the soil core for mass spectrometry were taken at the start (day 1) and end of the incubation (day 3 or 4) so as to limit the removal of gas from the below core headspace. In addition, because of the ability to account for the gas pool from above the core headspace and SF_6_ diffusion, a high temporal resolution was not required for pool dilution calculations. A total of 4% of the volume of gas in the headspace below the soil core was removed for analysis across the incubation period, which was factored into the gas concentration calculations. Headspace above the core were sampled (via the sampling tube) for SF_6_ and mass spectrometry (duplicate) analysis daily, with these always taken before headspace below the soil core samples. This was done by disconnecting the sampling tube (see Figure 1) from the headspace (to avoid creating negative pressure in the system and turbulent mixing with ambient air) and then connecting a syringe to the tube and taking samples before re‐connecting the sampling tube. The volume of the sampling tube (53.4 mL) was sufficient to collect two samples (maximum of 24 mL) without diluting with ambient air, as was tested (Supplementary Information S2, Figure S2).

One of the two duplicate samples was analysed by analysed for N_2_O and N_2_ concentration and enrichment using a Sercon trace gas analyser and Sercon 20:22 isotope ratio mass spectrometer (Sercon Ltd., Crewe, UK), whereas the other was spare in case of analysis failure. Samples were stored for 8 months before analysis due to COVID‐19 related restrictions to laboratory access and delays. Simultaneously, 12 mL N_2_O standards (5 ppm; *n* = 15) were stored with the samples to track any loss of concentration across the storage period. After this period, the mean standard concentration of this stored 5 ppm standard was 4.34 ppm ± 0.07. The analysed concentrations were adjusted to compensate for losses during storage.

For the analysis of SF_6_, the 10 mL samples were used to flush and fill a 1 mL loop that was then injected directly into a Shimadzu GC‐8A (Shimadzu KK, Kyoto, Japan) equipped with an Electron Capture Detector (ECD) and adapted for the rapid and precise analysis of SF_6_ in either the gas or water phase (Law et al., 1994). Separation of SF_6_ from O_2_ and N_2_O was achieved by a 3 m by 1/8″ stainless steel column packed with molecular sieve 5A. The system was calibrated daily using a six‐point calibration curve to cover the large range of concentrations observed between the two gas reservoirs. Analytical precision was typically better than 1%, and the detection limit was close to 2 pptv.

### 2.6 Diffusion coefficient ( *D*_s_ ) calculation

The natural logs of SF_6_ concentration depletion in the vessels were plotted against time for each WFPS treatment and soil depth. The diffusion coefficient (*D*_s_) was then calculated from the gradient of the depletion curve using Equation (3).

(3) $$C=\frac{2h\;\exp\left(-D_{s}\;a_{1}^{2}\;t/\varepsilon \right)}{L\left(a_{1}^{2}+h^{2}\right)+h},$$

where, *C* is the concentration of gas in the chamber (g m^−3^); *ε* is total air‐filled porosity (m^3^ of air m^−3^ soil); *L* is the depth of the soil core (m); *t* is time (h); *h* = *ε*(*aε*_c_), where *ε*_c_ = 1, is the air content of the chamber (m^3^ of air m^−3^ chamber); *a* is the volume of the chamber per area of soil (m^3^ of air m^−2^ soil). A plot of ln*C* against time becomes linear with slope –*D*_s_ *a*_*1*_^*2*^ *t*/*ε* for sufficiently large *t*. The value of *a*_1_ can be found using the table in Rolston and Moldrup (2002). The relative diffusion (*D*_s_/*D*_0_) of gas was calculated using the diffusion rate of SF_6_ in air, *D*_0_ (0.093 m^2^ s^−1^; Rudolph et al., 1996).

### 2.7 ^15^N‐N_2_O pool dilution calculation

The calculation of gross production and consumption of N_2_O was done using the modified (Wen et al., 2016, 2017) ^15^N‐N_2_O pool dilution method developed by Yang et al. (2011) from von Fischer and Hedin (2002):
(4)

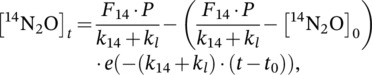



(5)

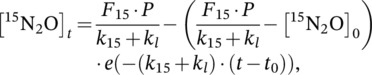

where the concentration of ^14^N_2_O at time *t* ([^14^N_2_O]_*t*_) is calculated as the product of the N_2_O concentration (ppb) and the ^14^N‐N_2_O atom% (i.e., 100 − ^15^N‐N_2_O atom%); [^15^N_2_O]_*t*_ is the concentration of ^15^N_2_O at time *t*, calculated as the product of the N_2_O concentration (ppb) and the ^15^N‐N_2_O atom% excess (assuming a ^15^N isotope composition of background N_2_O of 0.3688 atom%; Yang et al., 2011); *F*_14_ and *F*_15_ are the ^14^N_2_O (0.997) and ^15^N_2_O (0.003) mole fractions of emitted N_2_O, respectively; *k*_14_ and *k*_15_ are the first‐order rate constants of ^14^N_2_O and ^15^N_2_O reduction to N_2_, respectively, calculated using Equation (6) and the average literature value (*α* = 0.9924 ± 0.0036; Yang et al., 2011) for the stable N isotopic fractionation factors defined as *α* = *k*_15_ */k*_14_; *k*_l_ is the first‐order exponential decay constant for SF_6_ concentrations over time and represents physical loss via diffusion and/or advection (von Fischer & Hedin, 2002), calculated using Equation (6); *t* is the time (h) when the headspace was sampled. The gross N_2_O emission (ppb h^−1^), *P*, was calculated as the sum of Equations (4 and 5) relative to their mole fractions, solved using MATLAB (MathWorks, Version R2022a, USA).

The first‐order rate constants for ^15^N_2_O (*k*_15_) and SF_6_ (*k*_l_) were calculated using the following equation:

(6) $$k=-\frac{\ln\left(\frac{C_{t}}{C_{0}}\right)}{t},$$

where *k* is the first‐order rate constant; *C*_t_ and *C*_0_ are the concentrations (ppb) of the gas at sampling time *t* (h) and at *t* = 0, respectively. The rate constant for ^14^N_2_O, *k*_14_, was calculated by solving *α* = *k*_15_ */k*_14_, as described above.

The net N_2_O emission from the flow‐through small headspace was calculated as follows:

(7) $$F=t∙f∙\left(C_{\text{out}}-C_{\text{in}}\right),$$

where *F* is the flux (ppb h^−1^); *t* is the time (h) the sample is representative of; *f* is the flow rate of air through the headspace (L h^−1^) and *C*_out_ and *C*_in_ are the concentrations of N_2_O leaving and entering the headspace (ppb), respectively. The results from Equation (7) were then averaged and divided by the total incubation time to give a net flux (ppb h^−1^) per incubation vessel.

The net emission (Equation 7) and gross production (Equations 6 and 7) N_2_O rates were then converted to μg N kg^−1^ h^−1^ using Equation (8).

(8) $$F_{E}=\frac{F∙V_{h}}{10^{12}}∙\frac{p}{R∙\left(T+273\right)}∙\frac{28}{W_{d}}∙10^{9},$$

where *F*_E_ is either the net emission or gross production of N_2_O (μg N kg^−1^ h^−1^), *F* is the net or gross emission of N_2_O flux in ppb h^−1^; *V*_h_ is the headspace volume (L); *R* is the ideal gas constant (8.314 J K^−1^ mol^−1^); *p* is the pressure (Pa); *T* is the incubation temperature (°C) and 273 is the conversion constant to Kelvin; 28 is the molecular weight of N in N_2_O (g mol^−1^); *W*_d_ is the dry weight of the soil cores (g); 10^12^ and 10^9^ are unit conversion factors. Gross N_2_O consumption was then calculated as the difference between the gross N_2_O production and net N_2_O emission (Yang et al., 2011).

### 2.8 Statistical analysis

All data analyses were performed using R (R Core Team, 2017), with figures made using the R package ‘ggplot2’ (Wickham, 2016). Data were assessed for test assumptions by using the Shapiro–Wilk test (*p* > 0.05) for normality, and Levene's test for homoscedasticity (*p* < 0.05) as well as assessing the qqplots and the residual versus fitted plots. The difference in mean small headspace versus mean large headspace SF_6_ fluxes was tested with a Welch Two Sample *t*‐test. Differences in relative diffusivity were tested individually by depth and WFPS, using a Welch two‐sample *t*‐test. Difference in fluxes with depth and WFPS were tested using 2‐way ANOVAs. Data that did not meet assumptions were log or square root transformed to pass the Shapiro–Wilk and Levene's tests.

## 3 RESULTS

### 3.1 Relative diffusivity

**FIGURE 2:**
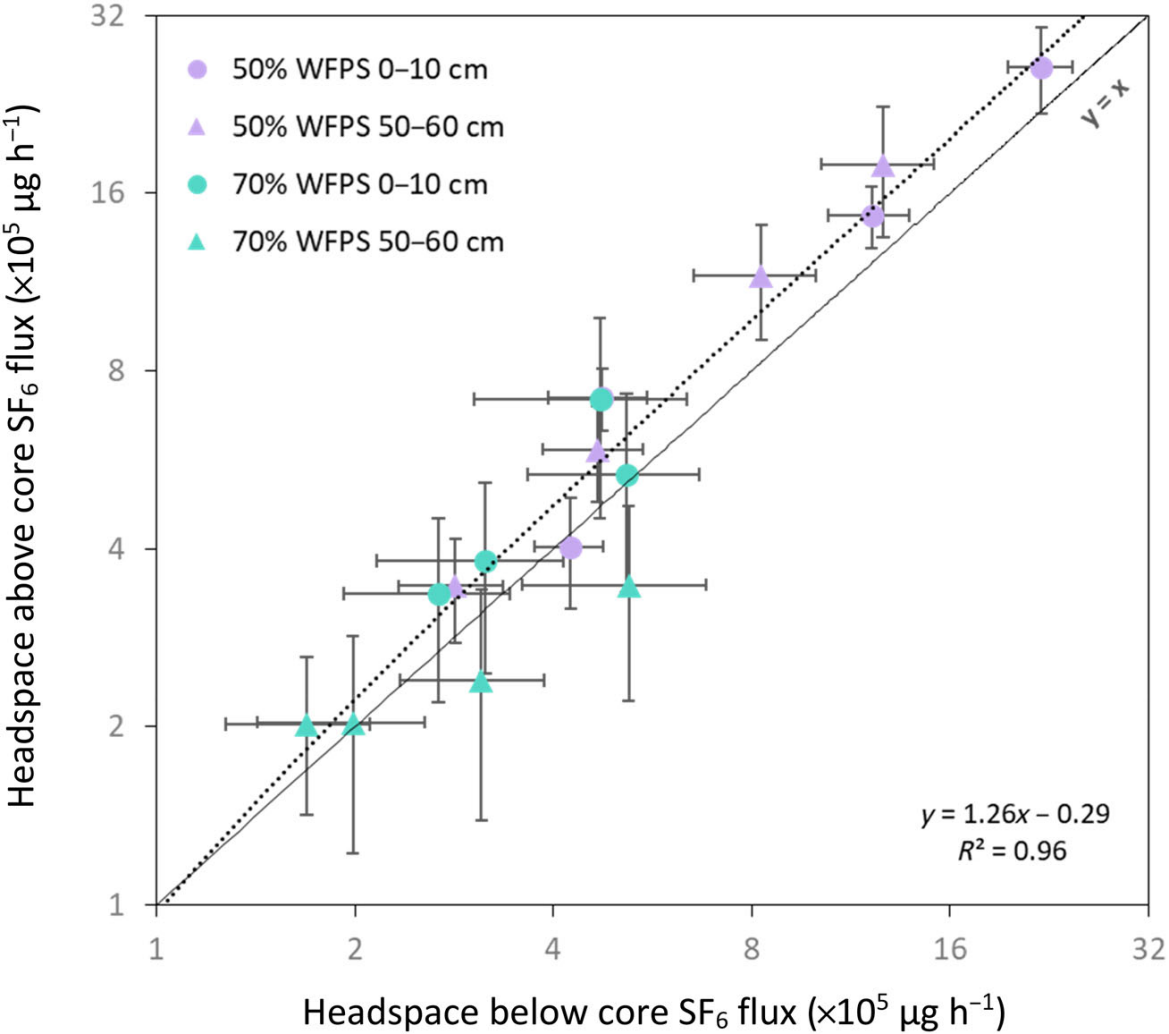
The fluxes of SF_6_ (means ± SEM; *n* = 6) from the headspace above versus the headspace below soil cores from the 0–10 and 50–60 cm soil depths at 50% and 70% water‐filled pore space. The dashed line represents the best fit for the flux data (*R* ^2^ = 0.96; *y* = 1.26*x* − 0.29) and the solid line represents the *y* = *x*. Note that the axes are logarithmic.

As a test to ensure the SF_6_ flux results from the small headspace and the depletion of SF_6_ from the large headspace corresponded with each other, the fluxes were plotted against each other (Figure 2). The proximity of the data to the *x* = *y* line demonstrate that they correspond well with each other. This is confirmed by the lack of a statistical difference between the fluxes from the small and large headspaces (*p* = 0.62). The linear trendline (*y* = 1.26*x* − 0.29) explained most of the variation in the data (*R* ^2^ = 0.96) but its deviation from the *x* = *y* line highlights that the mean measured headspace below the soil core flux was overall 16.3% lower than that measured in the headspace above the soil core. While the cores at 70% WFPS (*R* ^2^ = 0.55; *y* = 0.92*x* + 0.46) more closely aligned with the *x* = *y* 1:1 line, substantially more variation was explained by the line for the 50% WFPS cores (*R* ^2^ = 0.98; *y* = 1.21*x* + 0.54).

**FIGURE 3:**
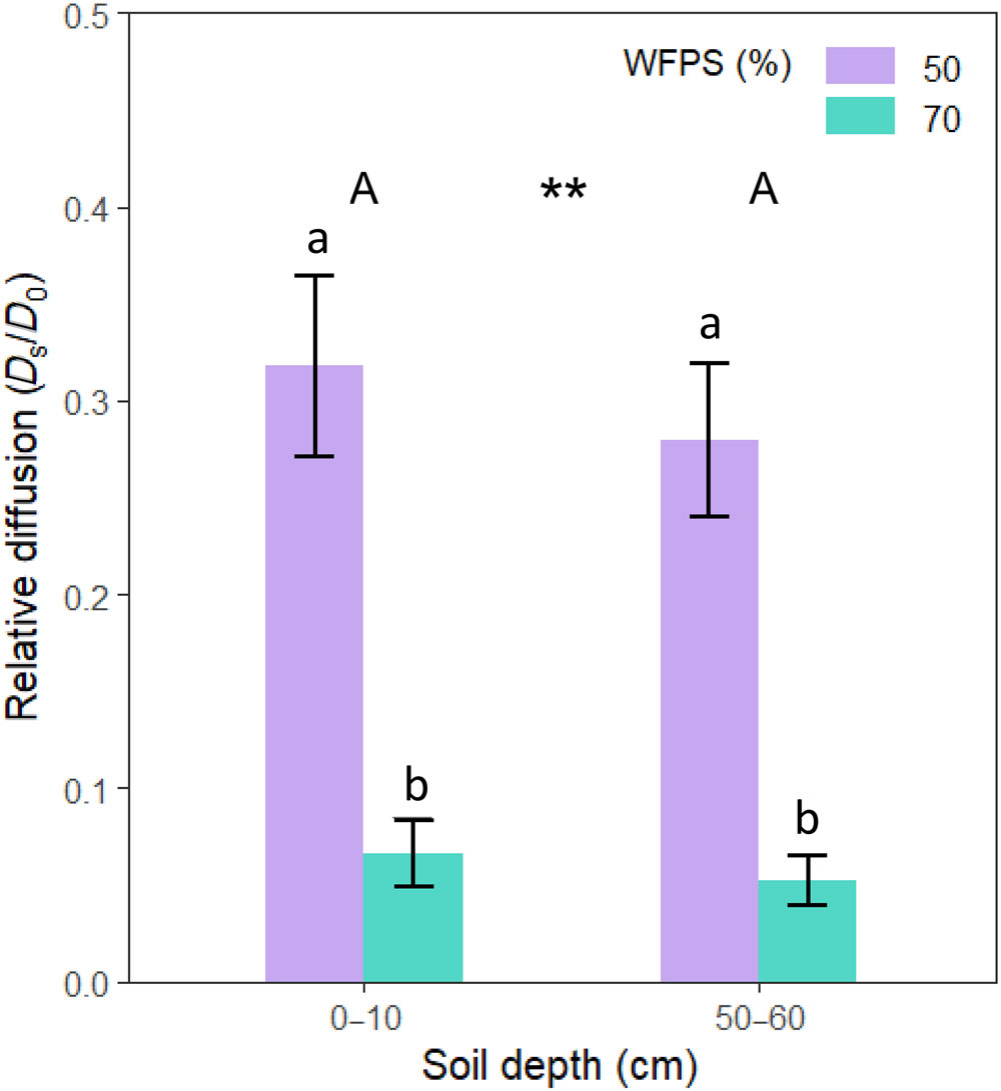
The mean (±SEM) relative diffusivity (*D*_s_/*D*_0_) of intact top‐ and subsoil cores at two different levels of water‐filled pore space (WFPS, %; *n* = 6). Different letters represent statistical difference of means between soil depths (upper‐case) and between soil depth and WFPS (lower‐case) at *p* < 0.05. Asterisks represent statistical difference in overall WFPS means at *p* < 0.001 (***); *p* < 0.01 (**); *p* < 0.05 (*) and *p* > 0.05 (‐).

The differences in relative diffusivity (*D*_s_/*D*_0_) in the top‐ and subsoil cores at 50% and 70% WFPS can be seen in Figure 3. In the 0–10 cm depth cores, the diffusivity was significantly lower (79% lower; *p* < 0.001) at 70% WFPS than when incubated at 50% WFPS. A similar trend was found for the 50–60 cm depth soil cores, where the diffusivity was significantly lower (81% lower; *p* < 0.001) at 70% WFPS than when incubated at 50% WFPS. Thus, the overall effect of WFPS on gas diffusivity was significant (*p* < 0.001), while depth the core was taken from was not. While the 50–60 cm cores did have 12% and 21% lower relative diffusivities compared to the 0–10 cm cores at 50% and 70% WFPS, respectively, these differences were not significant (*p* = 0.54).

### 3.2 Gross N_2_O emission and uptake

**FIGURE 4:**
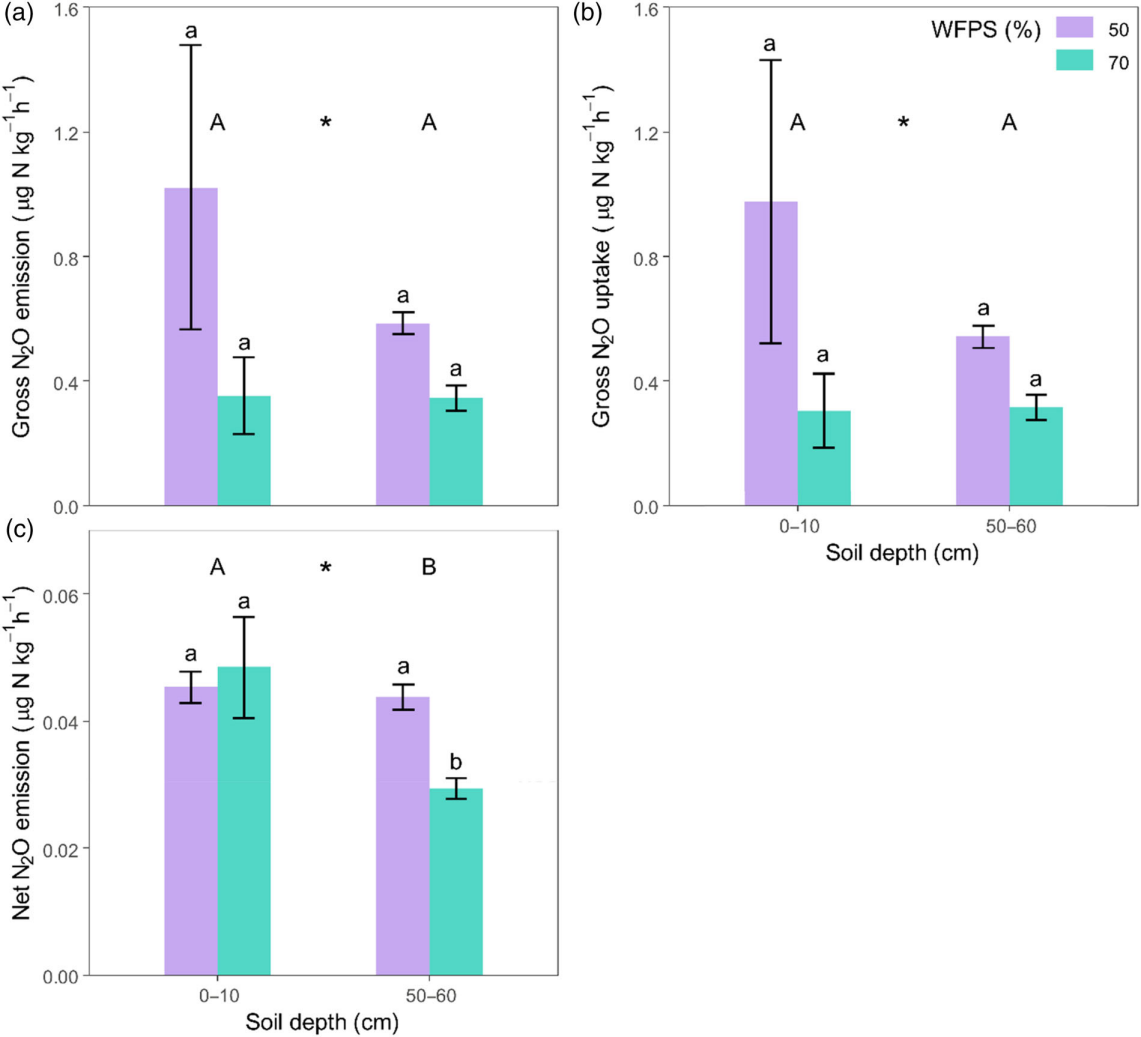
The gross N_2_O emission (i); gross N_2_O uptake (ii), and; net N_2_O emission (iii) (means ± SEM; *n* = 6) in intact 0–10 and 50–60 cm soil cores at 50% and 70% water‐filled pore space (WFPS) measured by the ^15^N‐N_2_O pool dilution method. Different letters represent statistical difference of means between soil depths (upper‐case) and between soil depth and WFPS (lower‐case) at *p* < 0.05. Asterisks represent statistical difference in overall WFPS means at *p* < 0.001 (***); *p* < 0.01 (**); *p* < 0.05 (*) and *p* > 0.05 (‐).

The 0–10 cm depth soil cores produced 186% more gross N_2_O at 50% WFPS (1.03 ± 0.46 μg N kg^−1^ ha^−1^) than at 70% WFPS (0.36 ± 0.12 μg N kg^−1^ ha^−1^). Similarly, the 50–60 cm depth cores produced 69% more gross N_2_O at 50% WFPS (0.59 ± 0.04 μg N kg^−1^ ha^−1^) than at 70% WFPS (0.35 ± 0.04 μg N kg^−1^ ha^−1^). As such, the overall effect of WFPS on gross N_2_O production was significant (*p* = 0.028; Figure 4a). However, the overall effect of soil depth on gross N_2_O production was not significant (*p* = 0.70), despite the 0–10 cm depth cores (0.69 ± 0.29 μg N kg^−1^ ha^−1^) producing 47% more gross N_2_O than the 50–60 cm cores (0.47 ± 0.04 μg N kg^−1^ ha^−1^), overall. This was driven by differences between the 50% WFPS cores at different depths, as there was only a 2% difference in gross N_2_O production between the depths at 70% WFPS. For gross N_2_O uptake, 216% more N_2_O was taken up in the soil at 50% (0.98 ± 0.46 μg N kg^−1^ ha^−1^) WFPS than at 70% WFPS (0.31 ± 0.12 μg N kg^−1^ ha^−1^) in the 0–10 cm soil cores. Following a similar trend in the 50–60 cm cores, 69% more N_2_O was taken up in the soil at 50% WFPS (0.54 ± 0.03 μg N kg^−1^ ha^−1^) than at 70% WFPS (0.32 ± 0.04 μg N kg^−1^ ha^−1^). The overall effect of WFPS on gross N_2_O uptake was significant (*p* = 0.036; Figure 4b). There was only a 4% difference in gross N_2_O uptake between the depths at 70% WFPS, whereas 49% more N_2_O was taken up by the 0–10 cm soil cores (0.64 ± 0.29 μg N kg^−1^ ha^−1^) compared to the 50–60 cm cores (0.43 ± 0.04 μg N kg^−1^ ha^−1^) at 50% WFPS. Despite this, there was no overall effect of soil depth on gross N_2_O uptake (*p* = 0.97).

### 3.3 Net N_2_O emission

Net emissions of N_2_O were overall higher in the cores at 50% WFPS (0.05 ± 0.01 μg N kg^−1^ ha^−1^) than at 70% (0.04 ± 0.001 μg N kg^−1^ ha^−1^, *p* = 0.042). This difference was driven by the 41% lower emissions from the 70% cores at 50–60 cm (0.03 ± 0.002 μg N kg^−1^ ha^−1^; Figure 4c) compared with the 0–10 cm cores at the same WFPS (0.05 ± 0.01 μg N kg^−1^ ha^−1^). In the 50–60 cm cores, the emissions from the 50% WFPS (0.04 ± 0.002 μg N kg^−1^ ha^−1^) treatment were 52% higher than in the 70% WFPS (0.03 ± 0.002 μg N kg^−1^ ha^−1^) treatment, but 5% lower than from the 70% WFPS cores. Overall, the 0–10 cm soil cores had 30% higher net N_2_O emissions (0.05 ± 0.01 μg N kg^−1^ h^−1^) compared to the deeper soil cores (0.04 ± 0.002 μg N kg^−1^ h^−1^; *p* = 0.014; Figure 4c).

## 4 DISCUSSION

### 4.1 Soil diffusivity

While the agreement between the small and large headspace SF_6_ fluxes was good (Figure 2), we attribute the overall higher fluxes in the headspace above the core compared to the headspace below the core is likely due to a technical factors. Because of the sampling of the headspaces above the cores prior to those below (to avoid any negative pressure influencing the above core headspace sample), there was a 1–2 h time delay between these, as the samples needed to be injected directly into the GC. Considering the exponential depletion of SF_6_ from the headspace below the cores, this time lag would translate to slightly different fluxes. Therefore, we believe the difference between the calculated fluxes is predominantly due to the delay in above core headspace samples. We believe the nature of the fit to be within an acceptable range of error for the relationship between the small and large headspaces to produce meaningful results from the ^15^N‐N_2_O pool dilution.

The relative diffusivity values in Figure 2 (0.024–0.480) are consistent with the expected values for the exponential increase in *D*_s_/*D*_0_ with increasing air‐filled pore porosity for soils with different overall pore architectures (Hashimoto & Komatsu, 2006) and using different measuring techniques (Allaire et al., 2008). The hypothesis that soil diffusion would be reduced by both increasing depth and WFPS was only partly confirmed (Figure 3). As expected, the highest WFPS in the soil reduced gas diffusivity of the soil substantially, but the different inherent physical soil characteristics (bulk density, porosity, and texture; Table 1) of the cores did not affect the *D*_s_/*D*_0_ of the soil when at the same WFPS. Fujikawa and Miyazaki (2005) found that *D*_s_/*D*_0_ increases with higher bulk density which they attributed to lower total porosity via the change in shape and size of pores which can be assumed to restrict gas movement, consistent with other studies (Balaine et al., 2013; Currie, 1984). However, these studies were all done on sieved and repacked soil which would create a more homogenous soil pore structure and can cause significant errors in determining the ‘true’ *D*_s_/*D*_0_ (Allaire et al., 2008). The inherent pore structure and preferential flow pathways (i.e., macropores, soil pipes and cracks) were preserved in the cores (although edge‐related diffusion was avoided by sealing these) and this heterogeneity is a primary factor driving gas flow and is very important for studying gas diffusion (Allaire et al., 2008; Chamindu Deepagoda et al., 2019; Guo & Lin, 2018). However, no difference in *D*_s_/*D*_0_ between intact soil cores at a range of depths, bulk densities and porosities have also been observed (Chamindu Deepagoda et al., 2019). We attribute this lack of difference between depths to the presence of natural macropores, pipes and preferential flow paths that create similarities in the diffusivity of gas through the soil and the differences in soil physical properties was not sufficient to drive differences in *D*_s_/*D*_0_.

### 4.2 Gross N_2_O uptake

Evidence for N_2_O consumption by soils is extensive in the literature (see review by Chapuis‐Lardy et al., 2007). In our study, we report gross N_2_O‐N uptake rates ranging from 0.03 to 2.79 μg N kg^−1^ h^−1^ (Figure 4b) which is a similar range to that measured by others in similar agricultural soils (Clough et al., 2006; Luo et al., 2022; Wen et al., 2016). N_2_O consumption rates, in our study, correlated closely with production rates, which is consistent with other studies (Wen et al., 2016; Yang et al., 2011; Yang & Silver, 2016), suggesting that consumption increased proportionally with N_2_O production (Figure 4a,b). These results uncovered a high potential for N_2_O uptake that would have been masked by higher N_2_O production had only the latter been measured.

The hypothesis that the uptake of N_2_O would be greater in the more microbially‐active topsoil compared to the subsoil was rejected (Figure 4b). While the uptake rate was highest in the topsoil cores at 50% WFPS, there was no statistical difference between depths. This is despite there being a lower microbial biomass (indicating size of the microbial community; Table 1) and a lower abundance of denitrification (*nirK*, *nirS*) and complete denitrification (*nosZ*) gene copies in the subsoil (indicating denitrification potential of the microbial community; Table S1). Scaling the magnitude of N_2_O uptake relative to the size and denitrification potential of the soil microbial community, it was much greater in the subsoil compared to the topsoil. Care should be taken with this interpretation as microbial biomass size does not necessarily indicate the activity of denitrifiers, and gene abundance does not necessarily link to process rates as discussed in a meta‐analysis conducted by Rocca et al. (2015).

The reduction of N_2_O to N_2_ can be considerable in the subsoil, dependent on a combination of inherent soil characteristics (C, NO_3_^−^) and physical conditions (WFPS, O_2_ concentration, diffusivity) (Clough et al., 1999, 2005; Semedo et al., 2020). Within the topsoil the organic C, total N, dissolved organic C, dissolved organic N and extractable NO_3_^−^ were greater than that found in the subsoil (Table 1). Thus, labile C and N substrate supply likely differed between depths during the course of the experiment. As discussed previously, NO_3_^−^ can outcompete N_2_O as the terminal electron acceptor during complete denitrification (Chapuis‐Lardy et al., 2007), potentially contributing to differences in N_2_O uptake rates between depths. In addition, the cores in this study were incubated at an O_2_ content similar to their in situ levels—which was 20.9% and 13% in the topsoil and subsoil incubations. Due to 38% less O_2_ in the subsoil cores, the formation of semi‐anaerobic and full anaerobic conditions required for N_2_O production and consumption would be more easily achieved. This is supported by others that found increased denitrification when O_2_ was restricted (Patureau et al., 1996; Schlüter et al., 2018), which would explain the lack of difference in gross N_2_O uptake between soil depths.

Higher WFPS decreases the diffusion of N_2_O produced in the soil to the surface and increases its residence time allowing for higher potential of complete denitrification of N_2_O to N_2_ (Balaine et al., 2013; Chamindu Deepagoda et al., 2019). While the diffusion rate did decrease with greater WFPS (Figure 3), this did not produce a difference between the N_2_O uptake rates of the soil cores incubated at different WFPS levels. In fact, the 50% WFPS cores had higher consumption rates. Therefore, we rejected our final hypothesis that N_2_O uptake would be higher with increasing WFPS.

N_2_O consumption is generally expected to occur under conditions of low N availability and high soil moisture (Chapuis‐Lardy et al., 2007). While there is extensive literature that suggests there is a high WFPS ‘critical threshold’ at which consumption predominantly takes place (ca. >60%–80%; Bateman & Baggs, 2005; Chamindu Deepagoda et al., 2019; Davidson, 1991), there are studies that have found no differences or even an increase in N_2_O uptake with lower WFPS (Goldberg & Gebauer, 2009; Khalil et al., 2002; Rosenkranz et al., 2006; Wu et al., 2013) and low N (Wang et al., 2018). A possible explanation for N_2_O consumption in drier soil is greater diffusivity allowing N_2_O present in air or headspace to diffuse to the denitrification site, where in the absence of NO_3_^−^, N_2_O may be used as an electron acceptor for denitrification (Chapuis‐Lardy et al., 2007). Bazylinski et al. (1986) demonstrated this in isolated denitrifier growth using only N_2_O as an electron acceptor. However, because of the presence of NO_3_^−^ in the top‐ and subsoil (Table 1) this is unlikely to contribute substantially. A possible alternative pathway is aerobic nitrate reduction, which is the bacterial reduction of NO_3_^−^ in aerobic conditions that can occur independently of denitrification gas‐producing reactions and is an underappreciated nitrate sink according to Roco et al. (2016). However, despite 84% more NO_3_^−^ in the topsoil compared to the subsoil (Table 1), no significant difference was measured between cores from these depths suggesting that this may not have been the primary mechanism. Without information on the changes in N pools it is not possible to determine the occurrence of this process. However, it suggests that substantial N_2_O consumption in our study could be driven directly and/or indirectly by aerobic processes rather than anaerobic denitrification processes (Wang et al., 2018; Wu et al., 2013). If this is the case and anaerobic microsites were not an important location for denitrification in this study, the calculated gross N_2_O production and consumption fluxes may be more accurate than expected from the pool dilution results. This is because the ^15^N‐N_2_O pool dilution method does not allow for accurate measurement of gross production and consumption of N_2_O in situations most likely to be occurring within anaerobic microsites. These are when (i) N_2_O produced is immediately consumed within the cells of denitrifiers, and (ii) produced N_2_O diffuses out of denitrifiers and is taken up by other microbes without mixing with the ^15^N_2_O label during the measurement period (Wen et al., 2016). Due to the 58% smaller volumes of the cores in this study compared to Wen et al. (2016), these processes may have been less likely to occur due to shorter diffusion distances reducing the time N_2_O spent in the soil and therefore the potential for its consumption in microsites.

### 4.3 Gross N_2_O emission

Gross N_2_O emission rates varied from 0.056 to 2.83 μg N kg^−1^ h^−1^ (Figure 4a), which is within the range of measurements reported in other studies (Clough et al., 2006; Luo et al., 2022; Wen et al., 2016). These rates may be low as N_2_O can be lost rapidly (hours) after wetting (Barrat et al., 2022; Smith & Tiedje, 1979). As the cores were brought to the desired WFPS ca. 18 h before the incubation, they may have already lost substantial soil N prior to incubation.

N_2_O production is driven by microbial denitrification and nitrification in the soil under partially anaerobic and aerobic conditions (Chapuis‐Lardy et al., 2007; Diba et al., 2011). The dominating process has been found to change from nitrification to denitrification at WFPS of 60%–70% (Bateman & Baggs, 2005; Pihlatie et al., 2004). This would suggest that the N_2_O produced in the 50% and 70% WFPS cores was predominantly from nitrification or denitrification, respectively. However, these may occur in the soil simultaneously (Bateman & Baggs, 2005; Pihlatie et al., 2004). Denitrification is a common source of N_2_O in many agricultural soils, and the close coupling between gross emission and uptake of N_2_O as found in this study (Figure 4a,b), suggests denitrification was the dominant process (Chapuis‐Lardy et al., 2007; Wen et al., 2016). According to Davidson (1991), N_2_O production is greatest when at or near field capacity (ca. 60% WFPS) as nitrification and denitrification rates are comparable sources of N_2_O occurring simultaneously. Therefore, a higher gross N_2_O emission in the soil cores at 50% WFPS could be explained by simultaneous denitrification and nitrification producing N_2_O. Nevertheless, we lack information to be able to source partition the N_2_O generated in this study. Recent advances in N_2_O isotopomer measurements are shedding light on microbial source partitioning of N_2_O, for example, Stuchiner and von Fischer (2022b) demonstrate denitrification was the predominant N_2_O production pathway in soils ranging from 50% to 95% WFPS and Harris et al. (2021) found that he proportion of N_2_O from denitrification did not decrease under even very low WFPS.

Gross emission rates were not different with depth in this study (Figure 4a). Emission rates of N_2_O have been observed to be higher in subsoil than in topsoil under certain conditions (Goldberg et al., 2008; Müller et al., 2004; Shcherbak & Robertson, 2019). This may be due to denser, deeper soils becoming anaerobic more quickly as a result of a restriction in diffusivity and lower pore volume (Berisso et al., 2013). As the subsoil cores were incubated with almost 38% less O_2_ than the topsoil, the formation of semi‐anaerobic and full anaerobic conditions required for N_2_O production would be more easily achieved. Therefore, despite higher biological N_2_O production potential in the topsoil (Table 1), it would suggest that physical N_2_O‐promoting conditions in the subsoil can match this potential.

### 4.4 Net N_2_O emission

Net emissions from the soil cores varied between 0.025–0.084 μg N kg^−1^ h^−1^ (Figure 4c). This low emission rate is expected from an unfertilized, low N arable soil (Table 1; Wen et al., 2016). The net N_2_O emission decreased with soil depth which is primarily due to the low rate from the 70% WFPS 50–60 cm cores (Figure 4c). This trend reflects the gross N_2_O uptake and emission in the soil, as the net emission is the gross consumption subtracted from the gross emission.

## 5 CONCLUSIONS

Using a novel dual‐headspace system for soil core incubation, we demonstrated that this method is reliable for measuring fluxes both above and below a soil core at controlled O_2_ concentration and for applying the ^15^N‐N_2_O pool dilution method. The fluxes measured from this system all fall within previously measured ranges measured in the field. We believe using a headspace both above and below the soil core is better than a single headspace approach as it is better placed to replicate the movement of gas through the soil and better mix gas from the reservoir with soil air, although this will require comparative testing. We provide evidence that the relative diffusivity of gas within intact soil cores does not differ with soil depth, likely because preferential flow pathways are preserved. This contrasts with studies that use sieved and repacked cores which allow for more equal mixing of labelled and non‐labelled isotope pools, but do not represent or measure true soil diffusivity. Gross N_2_O production and consumption rates did not differ with depth but were higher in the 50% WFPS cores. We attribute this to aerobic denitrification and simultaneous denitrification and nitrification for N_2_O consumption and production, respectively. We provide further evidence to challenge the hypothesis that only wet soils play a crucial role in N_2_O production, consumption, and net emissions. In addition, we challenge the notion that only soils with net negative emissions experience substantial N_2_O consumption rates. The results from this study provide a novel application of the ^15^N‐N_2_O pool dilution method and important evidence of N_2_O production and consumption fluxes in low‐N status, arable soil at different depths.

## AUTHOR CONTRIBUTIONS

Erik S. Button, Laura M. Cárdenas, David R. Chadwick, and David L. Jones conceived the study. Erik S. Button conducted the experiments and wrote the manuscript, with specialist technical support from Philip D. Nightingale and Elizabeth R. Dixon. Karina A. Marsden supported Erik S. Button with the pool dilution calculations and interpretation. All authors reviewed the manuscript.

## Supporting information


**Data S1.** Supporting Information.

## Data Availability

The data that support the findings of this study are available from the corresponding author upon reasonable request.
