# Supplementary material for: Separating N2O production and consumption in intact agricultural soil cores at different moisture contents and depths
Source: Eur J Soil Sci. 2023 Apr 27;74(2):e13363. doi: 10.1111/ejss.13363 (PMC10962597; doi:10.1111/ejss.13363)
Supplement: Supplementary file 1 — Data S1. Supporting Information. [file EJSS-74-0-s001.docx]

**Supplementary Information**

**Table S1**. The simplified equations of complete denitrification and nitrification reactions. The dashed line represents the transformation that does not occur during incomplete denitrification.

| Complete Denitrification | NO_3_^-^ NO_2_^-^  NO N_2_O N_2_ |
| --- | --- |
| Nitrification | NH_3_ NH_2_OH NO_2_^-^ NO_3_^-^  N_2_O N_2_O |

*S1. Incubation system specifications*
 The headspace chamber was made of an 83 mm x 60 mm Delrin^®^ rod (polyoxymethylene; Gilbert Curry Industrial Plastics Co Ltd., Coventry, UK) with the inside 53 mm bored out (Fig. 6.1). Stainless steel 3.18 mm and 6.35 mm Swagelok connectors (Swagelok Company, Solon, OH) connected the headspace with a 3.18 mm stainless steel and a 6.35 mm Teflon tube (a.k.a. polytetrafluoroethene; Context Pneumatic Supplies Ltd., Bolton, UK), respectively. A 3 m long 6.35 mm diameter Teflon sampling tube (53.4 ml) was connected to the headspace tube via 6.35 mm Swagelok union. This tube was tested against 3 other sampling approaches using a known concentration of standard N_2_O gas and was chosen as the most effective sampling option (Fig. S1 and Fig. S2). Silicone grease, applied in the joint between the core and the lid and between the headspace chamber and core, prevented any gas leakages (tested using a helium leak test). A fine nylon mesh placed on the underside of the core prevented any soil loss into the vessel throughout the incubation. A rubber septum (Hilltop Ltd., Warrington, UK) in the lid allowed for syringe application to, or sampling from, the vessels.


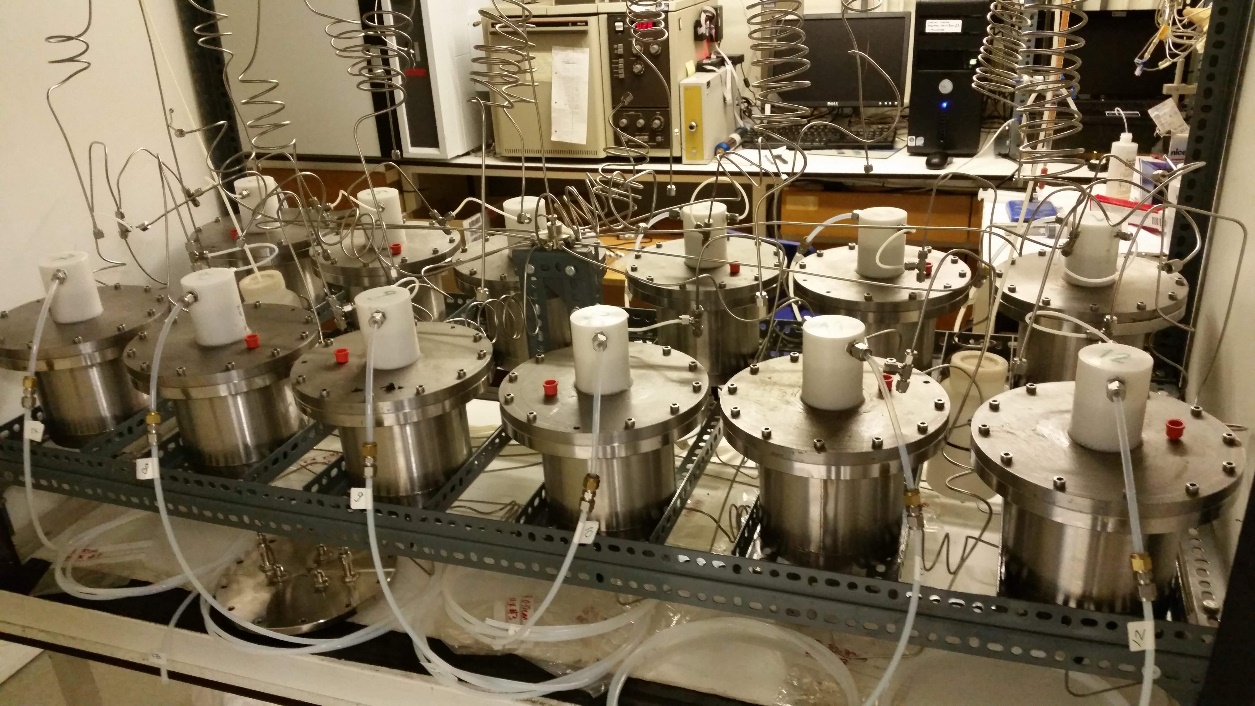
 **Fig. S1** Photograph of the incubation system used in the study.

*S2. Testing of different sampling methods*
Different sampling vessels were tested to determine the most effective method for evaluating gas sample quality. These were as follows:

1. ‘Tubing’: Samples from a sampling tube (53.4 ml) were taken by disconnecting the sampling tube from the standard gas flush and then connecting a syringe to the tube and taking 10 ml of gas, removing the syringe and reconnecting the sampling tube.
2. ‘Tank Open’: A sampling tank (150 ml) was disconnected from the standard gas flush, a syringe was attached, and a sample was taken. The valves remained in the open positions and the tank was reconnected.
3. ‘Tank Closed’: A sampling tank (150 ml) was disconnected from the standard gas flush, a syringe was attached, and the valve on the opposite side of the tank was closed before a sample was taken. The valve was opened after the sample was taken and the tank was reconnected.
4.
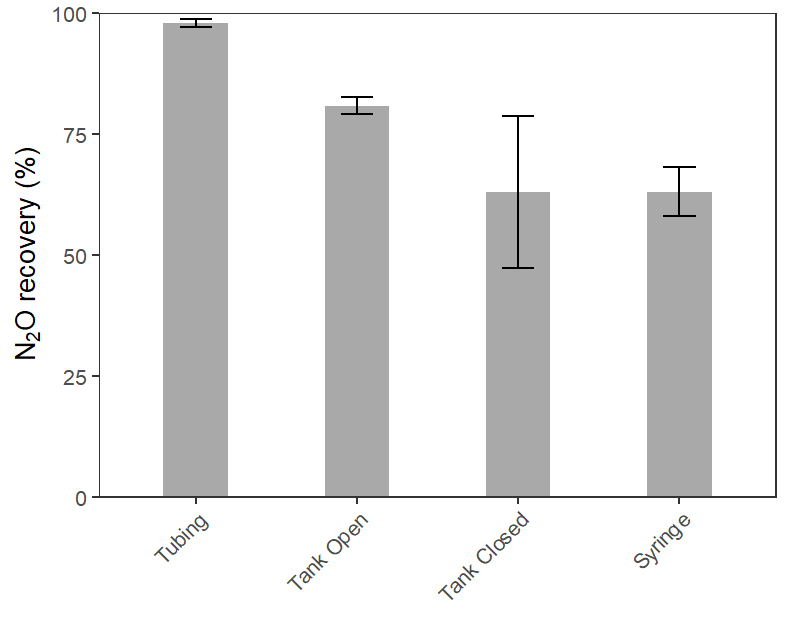
‘Syringe’: A 60 ml syringe without a plunger was connected to the standard gas flush and the plunger was carefully inserted and the syringe disconnected. After inserting the sample, the plunger was removed from the syringe and the syringe was reconnected.

**Fig. S2** Test of N_2_O recovery of a standard gas (i.e. 100% represents complete recovery) of different sampling methods. Bars are means ± SEM. The sampling receptacles were flushed for > 5 mins with a flow of approximately 100 ml min^-1^ of 100 ppm N_2_O standard gas before a syringe sample was taken (*n* = 6, except ‘Syringe’, where *n* = 3) and 10 ml analysed on a GC. From these results, it was decided that the ‘Tubing’ was the most effective sampling method.

**Fig. S3** Soil oxygen concentration choice for the core incubations at different depths. White points represent O_2_ data from a similar soil (Smith and Dowdell, 1974), while the ‘X’ represents the atmospheric concentrations (21%). The fitted line (polynomial; R^2^ = 0.99) was used to calculate the average oxygen concentration at the 3 depths the cores were extracted from (black points). These were 19.5% for the 0 - 10 cm, 15% for the 20 - 30 cm and 13% for the 50 - 60 cm cores, respectively. However, as the MFC is unreliable at low flow rate settings, it was decided that for the cores where low settings would be needed (0 – 10 cm cores) an ambient atmospheric O_2_ (i.e. 20.9 %) concentration was used instead. The horizontal grey bars show the depth range that the cores represent.


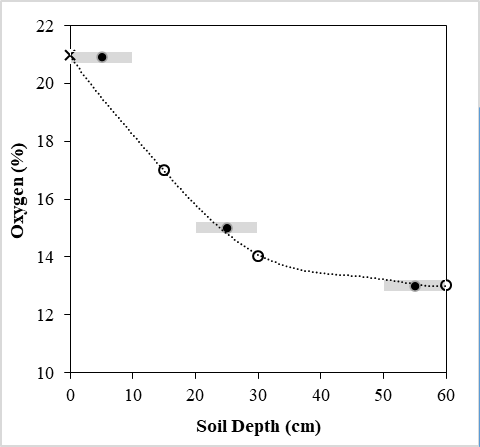


**Soil Oxygen Content (%)**

**Table S1**. Mean ± SEM (n = 4) gene abundances (*nirK*, *nirS*, *nosZ*) in the top- and subsoil of the soil used in this study. These were analysed by quantitative PCR, processed at the same time of sampling and following the methods described in de Sosa et al. (2018).

| Properties | Topsoil | Subsoil |
| --- | --- | --- |
|  | 0 – 10 cm | 50 – 60 cm |
| *nirK* gene (x10^8^ copies g^-1^) | 4.8 ± 0.4 | 0.9 ± 0.5 |
| *nirS* gene (x10^6^ copies g^-1^) | 6.9 ± 0.7 | 0.4 ± 0.2 |
| *nosZ* gene (x10^7^ copies g^-1^) | 5.3 ± 0.3 | 0.4 ± 0.3 |

*S3. Incubation of soil cores for drying*

While it could be argued that drying the soil cores at 40°C would alter the microbial community, no systematic differences in those which were dried at 40°C and those which were not, were observed. In addition, following the drying the cores were kept at room temperature overnight and a further 18 h in the acclimatisation period before the sampling began. Therefore, we conclude that the microbial community was not altered by soil drying at 40°C enough to induce differences in the N-related processes.


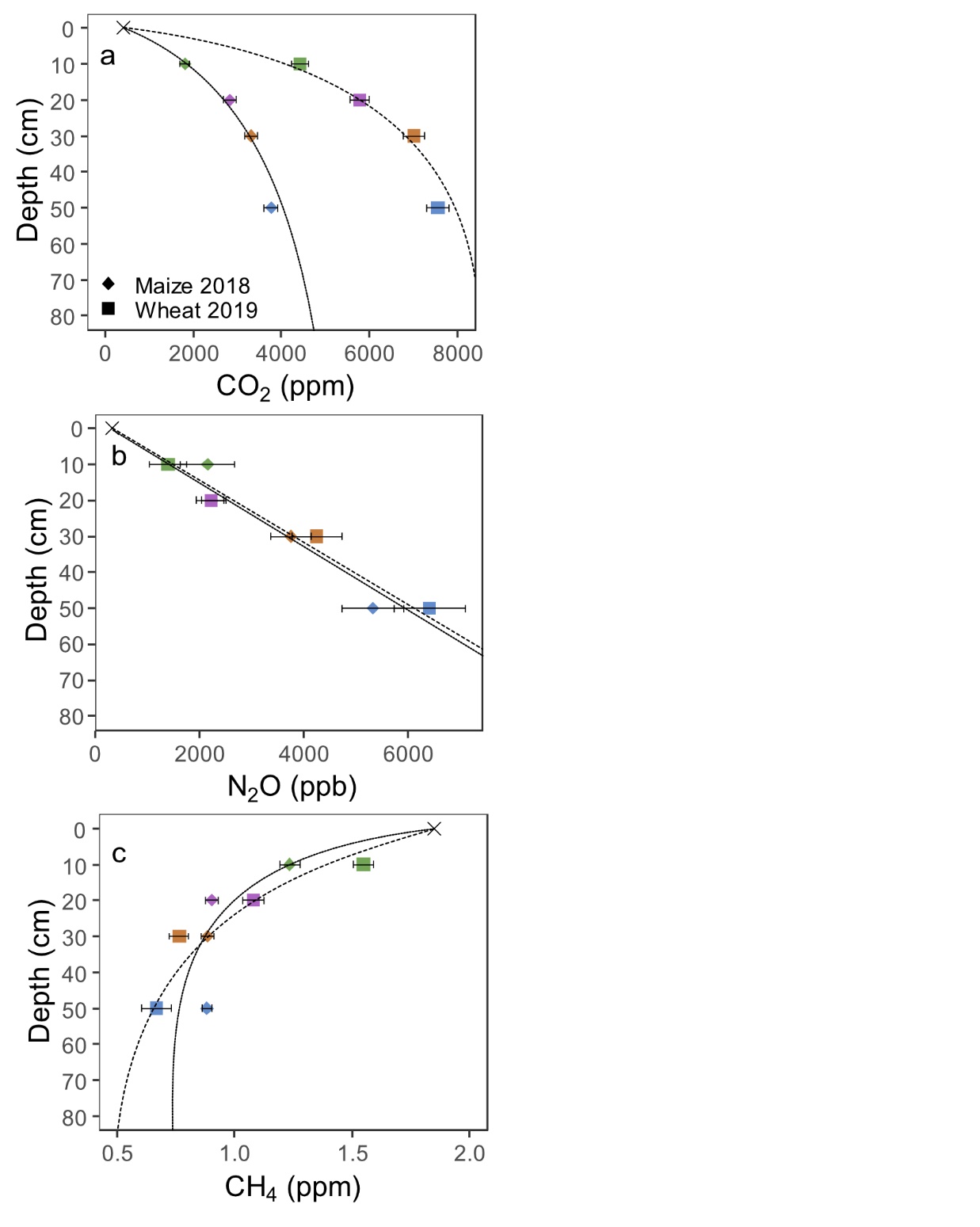

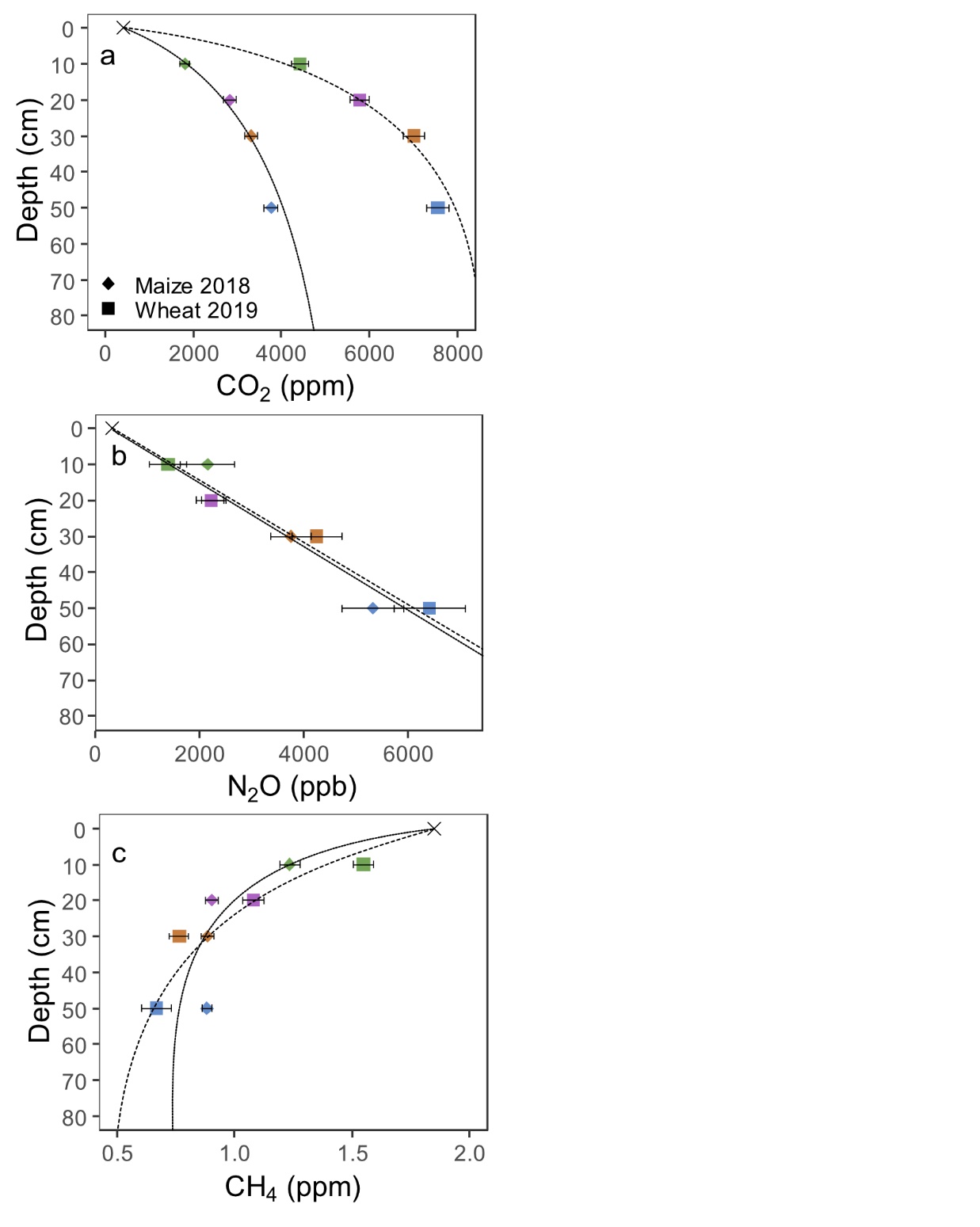


**Fig. S4** Depth profiles of mean (± SEM) gas concentrations of N_2_O from weekly sampling of gas collectors installed at different soil depths (*n* = 8) in a field under maize in 2018 (22 Jun 2018 – 19 Sep 2018; *N* = 644) and wheat in 2019 (22 May 2019 – 5 Sep 2019; *N =* 533). Colours reflect different sampling depths. Solid and dotted lines represent the maize and wheat, respectively. The ‘X’ at 0 cm depth represents the approximate ambient levels of N_2_O (330 ppb). The curves were forced to intercept the y-axis (0 cm) at the aforementioned concentrations.

*References*

de Sosa, L.L., Glanville, H.C., Marshall, M.R., Williams, A.P., Abadie, M., Clark, I.M., Blaud, A., Jones, D.L., 2018. Spatial zoning of microbial functions and plant-soil nitrogen dynamics across a riparian area in an extensively grazed livestock system. Soil Biology and Biochemistry 120, 153-164.

Smith, K.A. and Dowdell, R.J., 1974. Field studies of the soil atmosphere: I. Relationships between ethylene, oxygen, soil moisture content, and temperature. Journal of Soil Science, 25, 217-230.
